# A Case of Pediatric Post-Traumatic Stress Disorder Presenting as Attention Deficit Hyperactivity Disorder: A Case Report

**DOI:** 10.7759/cureus.1239

**Published:** 2017-05-10

**Authors:** Muhammad A Tahir, Nida R Gujar, Nusrat Jahan

**Affiliations:** 1 Psychiatry, Suny Upstate Medical University, Syracuse, NY; 2 Allied Hospital, Faisalabad, Punjab Medical College, Allied Hospital, Faisalabad, Pakistan; 3 Psychiatry, Mount Sinai Chicago

**Keywords:** post-traumatic stress disorder (ptsd), attention deficit hyperactivity disorder, pfeiffer syndrome, pediatric population

## Abstract

In the past few years, there has been increased recognition that children, who have faced traumatic incidences, can develop post-traumatic stress disorder (PTSD), just like in adults. We present a case of PTSD in a 6-year-old child who endured three surgical procedures because he was suffering from a congenital cranial stenosis (Pfeiffer) syndrome. Because of repetitive painful episodes, resulting from the syndrome, and then post-surgical complications, the child developed behavioral outbursts, hypervigilence, concentration problems, and irritability. In the past, the child was diagnosed with attention deficit hyperactivity disorder (ADHD) in the realm of his behavioral complaints, and he was already on stimulant medications for last one year. But there was no remarkable effect of pharmacotherapy on child’s behavior despite increasing dosages. Ultimately the child’s medical and psychiatric history was reviewed and a diagnosis of pediatric PTSD was made. Stimulant medications were discontinued and management was started on the lines of pediatric PTSD, resulting in a remarkable improvement in child’s psychiatric outcome.

## Introduction

The term post-traumatic stress disorder (PTSD) was first coined in Diagnostic and Statistical Manual of Mental Disorders (DSM) 3. The DSM-4 describes three symptom clusters in PTSD: persistent re-experiencing of the trauma (e.g., intrusive memories and flashback experiences, often triggered by exposure to traumatic reminders, and recurring trauma-related nightmares); avoidance of traumatic reminders (including places, people, and conversations) and a general numbing of emotional responsiveness; and chronic physiological hyperarousal, including sleep disturbances, poor concentration, and hypervigilance to threat. In order to meet the PTSD diagnosis, at least one re-experiencing symptom, three avoidance/numbing symptoms, and two hyperarousal symptoms should be present for at least one month, and must cause significant distress or functional impairment [1]. When symptom duration is less than one month, a diagnosis of acute stress disorder (ASD) is made [1]. Children who are suffering from chronic syndromes need multiple admissions, medical and surgical interventions, and sometimes on top of that they have to face complications. Multiple cycles of hospital admissions, therapies, and complications pose significant stressors to a child’s psychological health. In this article, we will describe a case report of a pediatric PTSD. Our main aim is to help the pediatricians in early diagnosing and managing the PTSD in pediatric population.

## Case presentation

A 6-year-old child with past history of cranial stenosis (Pfeiffer) syndrome and attention deficit hyperactivity disorder (ADHD) was referred to the psychiatric emergency of a tertiary care hospital in Faisalabad, Pakistan. Complaints at the presentation were worsening aggression and behavioral outbursts that had caused the patient to be picking at his sutures leading to dehiscence of the suture sites along with multiple episodes of sleep problems, increased arousal, exaggerated startle, hypervigilance, and behavioral reenactment for the past three years. Patient’s mother reported that there were many times when the child lost control and there had been many spells of worsening of such behavior especially when the child is brought to the hospital for the management of cranial stenosis syndrome. The patient had three surgeries in the past at the ages of 2, 3, and 6 years. The first surgical procedure was fronto-orbital advancements for bilateral coronal synostosis. Wound infection and septicemia developed as part of post-surgical complication and prolonged the hospital stay. The second and third surgical procedures were mainly dental interventions for overcrowding teeth. The patient was diagnosed with ADHD because of the behavioral symptoms and he was started on the Adderall and clonidine one year ago. Parents reported no significant improvement in the psychiatric symptoms. On examination, the child was very aggressive and did not engage well in the mental status and physical examinations. We reviewed the patient’s history of ADHD under the DSM-5 criteria and found that the child is not fulfilling the entire criteria. So we ruled out ADHD and the history of pediatric PTSD was taken in the light of DSM-5 criteria, and the child fulfilled the criteria and so the management was started on the lines of pediatric PTSD. Stimulants and clonidine were gradually discontinued. Trauma-focused psychotherapy was started under the supervision of psychotherapist. Aggression episodes, hypervigilance, and sleep problems declined gradually over a period of three months. The child is no longer picking at his suture sites. Parents reported that whenever the child is brought to the hospital, there are episodes of increased arousal, but overall the behavioral health is much better as compared to earlier.

## Discussion

PTSD in children occurs when the child is exposed to a traumatic event such as injuries, accidents, death, physical and sexual violence, and recurrent painful episodes because of some diseases, its complications, or management. Prevalence is higher for female than male (8.0% vs. 2.3%) and increases with age [2]. Children with subthreshold criteria for PTSD demonstrate substantial functional impairment and distress [3]. An undiagnosed case of PTSD in children can have long lasting complications like major depression, aggression, substance abuse and dependence, suicides and physical comorbidities (chronic fatigue, fibromyalgia, irritable bowel syndrome) [4]. The American Psychiatric Association’s Diagnostic and Statistical Manual, Fifth Edition (DSM-5), lists the following diagnostic criteria for PTSD in adults, adolescents, and children older than 6 years [5]:

Exposure to actual or threatened death, serious injury, or sexual violence (any undesired sexual activity is sexual violence)

Presence of one or more specified intrusion symptoms in association with the traumatic event(s)

Persistent avoidance of stimuli associated with the traumatic event(s)

Negative alterations in cognitions and mood associated with the traumatic event(s)

Marked alterations in arousal and reactivity associated with the traumatic events(s)

Duration of the disturbance exceeding one month

Clinically significant distress or impairment in important areas of functioning

Inability to attribute the disturbance to the physiologic effects of a substance or another medical condition

DSM-5 criteria for PTSD in children aged 6 years or younger are as follows:

Directly experiencing the traumatic event, witnessing the event, or learning it occurred to a parent or caregiver

Intrusion symptoms associated with the event (recurrent memories, distressing dreams, dissociative reactions, marked distress, or physiological reaction in response to exposure to traumatic triggers)

Avoidance of situations or things that arouse recollections of the trauma OR negative alterations in cognitions (increased negative emotions, decreased interest in significant activities, social withdrawal, decreased positive emotions)

Alterations in arousal and reactivity associated with the traumatic events (two of irritability, hypervigilance, exaggerated startle, concentration problems, sleep disturbance )

Duration of the disturbance exceeding one month

Clinically significant distress or impairment in relationships with parents, siblings, peers, or other caregivers or in school behavior

Inability to attribute the disturbance to the physiologic effects of a substance or another medical condition

There are no specific laboratory tests for PTSD. Several psychometric measures, such as semistructured interviews or self-report measures, are used to evaluate PTSD in children. Child PTSD Symptom Scale (CPSS) is an effective scale with the sensitivity of 84% and specificity of 72% [6]. Management starts with the safety. The child should be in the safe environment. If there is suspicion of child abuse, child protective services (CPS) should be called. Non-pharmacological therapies in the form of trauma-focused cognitive behavioral therapy (CBT) and dialectical behavior therapy (DBT) are the preferred initial management strategy for PTSD in children. DBT, helping children and adolescents to deal with painful feelings, may be necessary before CBT can be done [7]. Currently, no selective serotonin reuptake inhibitors (SSRIs) are food and drug administration (FDA) approved for the treatment of PTSD in the pediatric population. But, in children with persistent symptoms despite psychotherapeutic interventions, pharmacologic treatment may be used. Pharmacologic agents that can be considered in this aspect are SSRIs though not FDA approved, alpha-adrenergic agonists (e.g., guanfacine and clonidine), and beta-blockers (e.g., propranolol).

It is not unusual that the symptoms of pediatric PTSD may mimic like ADHD. It is very important for the physicians to imply the ADHD DSM-5 diagnostic criteria comprehensively. DSM-5 criteria for the diagnosis of ADHD needs to fulfil the following sub-criteria: at least six or more symptoms either of inattention or hyperactivity for the period of six months, symptoms should be present prior to age of 12 years and must be in two different settings, symptoms should reduce the quality of life, and the symptoms are not better explained by other mental disorders. Just using the symptoms to diagnose ADHD and not paying attention to other factors like age, setting, quality of life affected, and excluding other psychiatric diseases may result in over-diagnosis of the ADHD. Stimulants have excellent results for ADHD in adolescents and good results in children. If in a child (like in this case), stimulants are not improving the symptoms of ADHD, then the health-care provider should review the diagnosis of ADHD.

## Conclusions

Pediatric population suffering from chronic and congenital anomalies is exposed to multiple painful episodes because of diseases, their complications, and management. Mental health physicians should have a low threshold for pediatric post-traumatic stress order in these circumstances.
